# Species Differences in Blood Lymphocyte Responses After Spinal Cord Injury

**DOI:** 10.1089/neu.2022.0122

**Published:** 2023-04-28

**Authors:** Carlos Ayala, Morgan Fishman, Margot Noyelle, Hamid Bassiri, Wise Young

**Affiliations:** ^1^ W.M. Keck Center for Collaborative Neuroscience, Rutgers, The State University of New Jersey, Piscataway, New Jersey, USA.; ^2^ New Jersey Medical School, Rutgers, The State University of New Jersey, Newark, New Jersey, USA.; ^3^ Children's Hospital of Philadelphia, University of Pennsylvania Perelman School of Medicine, Philadelphia, Pennsylvania, USA.

**Keywords:** blood, infection, leukocyte, lymphocyte, spinal cord injury, spinal cord injury-induced immune depression syndrome

## Abstract

People with spinal cord injury (SCI) get recurrent infections, such as urinary tract infections (UTIs) and pneumonias, that cause mortality and worsen neurological recovery. Over the past decades, researchers have proposed that post-SCI lymphopenia and decreased lymphocyte function increase susceptibility to infections and worsen neurological outcome in humans, leading to a condition called SCI-induced immune depression syndrome (SCI-IDS). In this review, we explore how SCI affects blood lymphocyte homeostasis and function in humans and rodents. Understanding how SCI affects blood lymphocytes will help the management of recurrent infections in spinal cord injured people and shed light on the clinical translation of findings in animal models to humans.

## Introduction

Spinal cord injury (SCI) causes disabling neurological deficits^1^ in people worldwide.^2,3^ The most common causes of SCI are motor vehicle accidents in young adults^4^ and falls in the elderly.^5^ People with SCI are more prone to develop bacterial infections in the respiratory^6,7^ and urinary tracts,^8^ and in pressure ulcers.^9–11^ A higher infection incidence^12–15^ leads to complications, such as sepsis,^16^ worsens recovery rates,^17–19^ and increases mortality.^13,20–23^

People with SCI may get more infections for several reasons. First, clinical management of respiratory function,^24^ urinary catheterization,^25^ and immobility^25–28^ increases infection risk. Second, researchers have proposed that SCI causes an “immune depression syndrome” (IDS),^29,30^ “immune dysfunction,”^31^ or “immune deficiency,”^32–34^ which makes this patient population more susceptible to bacterial infections.

Leukocytes are immune cells that fight infectious pathogens and repair damaged tissue, including the spinal cord after SCI.^35^ In adults, immune cells arise from hematopoietic stem cells (HSCs) in the bone marrow.^36,37^ HSCs make common myeloid progenitor cells and lymphoid progenitor cells that differentiate into granulocytes and monocytes, and lymphocyte subpopulations, respectively.^36^ Leukocytes have been studied in the last two centuries because clinical blood collection is routine, and blood cells can be quantified per milliliter of blood.

Myeloid-derived neutrophils and monocytes quickly encircle infections and damaged tissue.^35,36^ Myeloid-derived cells have membrane-bound major histocompatibility complexes that present pathogen or self-derived peptide fragments (antigens) to lymphocytes.^36^ Antigen recognition leads to lymphocyte differentiation into antigen-specific, mature T and B cells that provide long-lasting immunity in case of reinfection.^36^

The propensity for recurrent infections in people with SCI^12–15^ led researchers to explore how SCI affects systemic immune responses in both humans and rodents. SCI initiates a systemic immune response, increasing pro-inflammatory cytokines^38–41^ and catecholamine concentrations,^42,43^ that causes neutrophils to demarginate in the bloodstream^44–48^ and squeeze leukocytes from the spleen and lymphatic organs.^42,43^ The consequent increase in circulating neutrophils lasts for several days and then returns to baseline.^44–48^ However, the effect of SCI on blood lymphocyte function appears to be species-dependent.^42,43,49–55^

All leukocyte populations enter the injured spinal cords of humans and rodents. Leukocytes arrive at the injured spinal cord during injury repair driven by microglia and astrocytes responding to tissue necrosis.^56–58^ Myeloid-derived cells help remodel neural tissue, as described in detail by Hawthorne and Popovich^59^ and David and colleagues.^58^ Lymphocyte infiltration is prominent in the injured spinal cords of rodents, with both severity and timeline depending on the animal model and species.^56,57,60–62^ Chang,^63^ Fleming and associates,^64^ and Zrzavy and co-workers^65^ showed that, compared with rodents, far fewer T and B cells enter the human injured spinal cord in both the acute and chronic phases of recovery.

The function of blood lymphocyte infiltration into the injured spinal cord remains controversial. Instead, much emphasis has been given to the effect of SCI on lymphocyte function in peripheral blood and lymphatic tissue. In this review, we explore the effects of spinal cord trauma on peripheral blood lymphocytes and the clinical relevance of rodent models for SCI-induced immune depression syndrome (SCI-IDS).

## Does SCI Cause Blood Lymphopenia in Humans?

### Effects of SCI on circulating T cells in humans

T and B cells are classically considered part of the adaptive immune response that helps fight reinfection from pathogens.^66^ T and B cells differentiate into long-term memory cells and proliferate in response to epitopes on pathogens or on antigen-presenting cells.^67^ People with T cell or B cell lymphopenia are less able to fight infections.^68^ Several groups^29,30,47^ hypothesized that injury-induced decreases in adaptive immune cells contribute to the higher incidence of recurrent infections in people with SCI. In 2009, Riegger and colleagues^47^ identified T cells using cluster of differentiation (CD)3+, a pan-T cell marker, and showed that blood samples from people with SCI have 80% fewer T cells within 24 h after injury than surgical controls. This decrease in T cells is inversely proportional to an increase in blood neutrophils in response to spinal cord trauma.^44,45,47^

The effect of SCI on chronic CD3+ T lymphocyte homeostasis in the blood is less understood. The acute inflammatory response during the first days after injury is overwhelming, with blood neutrophils first far outnumbering the number of lymphocytes.^44,45,47^ However, once blood neutrophilia dissipates, there is no consensus on how SCI affects circulating CD3+ T cells compared with other leukocytes. Campagnolo and associates^69^ reported that people with chronic complete cervical SCI have similar percentages of blood CD3+ T cells as age- and sex-matched, neurologically intact people. Yet, in another cohort study conducted by the same group,^70^ people with chronic SCI had higher percentages of blood CD3+ T cells. Iversen and co-workers^71^ reported that neither the number of blood T cells nor T cell lymphopoiesis change significantly in the chronic phase of injury. In contrast, Monahan and colleagues^72^ reported that people with chronic SCI have lower percentages of blood CD3+ T cells compared with individuals without a history of SCI. Finally, Riegger and colleagues^47^ showed that the number of blood CD3+ T cells initially decreases but then returns to normal 1 week after SCI and remains stable thereafter.

Helper CD4+ T cells and cytotoxic CD8+ T cells are the most commonly studied CD3+ T lymphocytes. CD4+ T cells activate macrophages to remove bacteria, recruit neutrophils,^73^ and present antigens to B cells to induce clonal expansion of antibody-secreting plasma cells.^74^ Low numbers of helper CD4+ T cells, such as in people with human immunodeficiency virus (HIV), lead to higher risk for recurrent infections, especially by opportunistic pathogens.^75^ CD8+ T cells attach to virus infected cells to initiate cell lysis and secrete cytokines that activate phagocytes which ingest viral epitopes for antigen presentation.^76^ Antigen recognition by B cells then causes clonal expansion of antibody-secreting cells against that virus.^76^

A decrease in the number of circulating CD4+ and CD8+ T cells may increase the risk for infections in people with SCI. Kyritsis and co-workers^77^ determined the proportion of each leukocyte in the blood with gene expression after SCI. Compared with healthy individuals, people with SCI had a lower proportion of CD4+ related gene expression.^77^ However, there are few studies quantifying the number of blood lymphocyte subpopulations in the first days after SCI.

Controversy exists regarding the effect of SCI on blood CD4+ T cells in the chronic phase of recovery. Campagnolo and associates^70^ used flow cytometry to show that the percentage of blood CD4+ T cells increases significantly in people with chronic SCI. In another cohort, Monahan and colleagues^78^ showed that people with chronic SCI have lower percentages of blood CD4+ T cells than uninjured individuals. Interestingly, a greater proportion of CD4+ T cells were human leukocyte antigen-DR isotope (HLA-DR)+ in the group with SCI, suggesting CD4+ T cell activity is higher in people with SCI than uninjured people.^79^ However, Herman and co-workers^80^ reported no significant changes in the proportion of blood CD4+ T cells in people with chronic SCI rostral to T5 compared with uninjured controls.

There is more agreement on the chronic effect of SCI on blood CD8+ T cells. Neither Monahan and colleagues^72^ nor Herman and co-workers^80^ found significant changes in the percentage of circulating CD8+ T cells in chronic spinal cord injured people.

People with significantly lower T cell counts per milliliter of blood are more prone to infections. The absolute number of blood lymphocytes is used as a clinical indicator to determine infection risk in immunocompromised patients.^81^ Because the ratio of circulating lymphocytes to neutrophils changes drastically after SCI,^44,45,47^ future SCI studies must include changes in both percentages and absolute cell counts to better understand how damage to the spinal cord affects circulating immune cell homeostasis.^82^

Conflicting findings on the effect of SCI on long-term blood T cell homeostasis may have occurred from differences in methodology used to quantify blood lymphocytes. Some teams reported T cells as percentages or proportions of gene expression, whereas others quantified absolute counts (Table 1). Although a Ficoll density gradient can separate granulocytes from mononuclear cells, sample handling affects viability and the number of lymphocytes available for flow cytometry.^83^ Quantifying the number of T cells per milliliter of blood, as is commonly done in people with HIV, is the most effective method to assess immune function in immunocompromised people.^75^ Because there is an inverse relationship between blood neutrophils and lymphocytes during the first week after SCI,^44,45,47^ it would be more helpful to understand how neurotrauma affects blood T cell populations in absolute numbers.

**Table 1.** Manuscripts Comparing Blood Lymphocyte Responses in People After SCI

Author	Subjects and injury type	Mean time after SCI	Cell type	Hematological methods	Results
Campagnolo et al. 1994	5 people with complete cervical SCI (4 men, 1 woman (mean age = 36.2 years), 5 controls (4 men, 1 woman, mean age = 35.1 years)	33.8 months	T cells, helper T cells, suppressor T cells, B cells, markers not indicated	Flow cytometry	T cells • Control: 76.02% • SCI: 81.18% Helper T cells: suppressor T cells • Control: 1.82 • SCI: 2.26 B cells • Control: 12.96% • SCI: 14.0%
Iversen et al. 2004	6 tetraplegic and 6 paraplegic men (aged 24-42 years) with complete SCI and 6 age-matched controls	15.7 years	CD4+ helper T cells, CD8+ cytotoxic T cells, CD19+ B cells	Microarray gene expression profiling	Helper T cell, cytotoxic T cell, and B cell related gene clusters and modules
Campagnolo et al. 2008	36 people with SCI. Injuries spinal level T6 and above, *n* = 26 (23 men, 3 women, mean age = 37.7 years). Injuries spinal level T6 and below, *N* = 10 (mean age = 35.6 years), all with complete injuries and 34 age-matched men (mean age = 36.4 years).	13 months for T6 and above, 41 months for T6 and below	CD3+ T cells, CD3+CD4+ helper T cells, CD3+CD8+ cytotoxic T cells, CD19+ B cells	Flow cytometry	T cells^a^ • Control: 72% • SCI: 75% Helper T cells^a^ • Control: 46% • SCI: 48% Cytotoxic T cells^a^ • Control: 26% • SCI: 25% B cells^a^ • Control: 16% • SCI: 15%
Riegger et al. 2009	Injuries between spinal level C4 and L1, *n* = 16, (mean age = 38 years), 10 age-matched controls	<24 h, 3-4 days, 6-8 days, 25-30 days, and 105-136 days after SCI	CD3+ T cells, CD19+ B cells	Flow cytometry	T cells^b^ • Control: 1481 • <24 h: 297 • 3-4 days: 597 • 6-8 days: 1652 • 25-30 days: 1966 • 105-136 days: 1771 B cells^b^ • Control: 256 • <24 h: 72 • 3-4 days: 133 • 6-8 days: 442 • 25-30 days: 373 • 105-136 days: 224
Monahan et al. 2015	SCI between spinal level C1 to L5, *n* = 22, (mean age = 56 years), 11 controls (mean age = 50 years)	17 years	CD3+ T cells, CD3+CD4+ helper or CD3+CD8+ cytotoxic T cells + HLA-DR; CD3+CD4+CD25+CD 127lo and CCR4+, HLA-DR+, or CCR4+HLA-DR+ regulatory T cells	Flow cytometry	T cells • Control^a^: 60% • SCI: 56.5% Helper T cells • Control^a^: 74% • SCI: 68.4% of T cells activated helper T cells • Control^a^: 1.6% • SCI: 1.61% of CD4+ cells Cytotoxic T cells • Control: 23 ± 2% of T cells • SCI: 19 ± 2% of T cells activated cytotoxic T cells • Control: data not shown • SCI: 1.71% of CD8+ cells Regulatory T cells • Control^a^: 6% of CD3+CD4+ cells • SCI^a^: 7% of CD3+CD4+ cells regulatory T cells that are CCR4+: • Control^a^: 38% of CD3+CD4+/ CD25+CD127lo cells • SCI^a^: 55% of CD3+CD4+/ CD25+CD127lo cells regulatory T cells that are HLA-DR+: • Control^a^: 7% of CD3+CD4+/ CD25+CD127lo cells • SCI^a^: 12% CD3+CD4+/CD25+CD127lo cells Regulatory T cells that are CCR4+HLA-DR+: • Control^a^: 5% of CD3+CD4+/ CD25+CD127lo cells • SCI^a^: 8% of CD3+CD4+/CD25+CD127lo cells
Herman et al. 2018	SCI at spinal level T5 and above, *N* = 31, (25 men, 6 women, mean age = 55 years). Controls, *n* = 26, (19 men, 7 women, mean age = 48 years)	15.7 years	CD4+ helper T cells, CD8+ cytotoxic T cells, CD19+ B cells	Microarray gene expression profiling	Helper T cell, cytotoxic T cell, and B cell related gene clusters and modules
Kyritsis et al. 2021	People with SCI, *n* = 38. People with non-CNS injuries, *n* = 10. People without an injury, *n* = 10.	Hyperacute post-SCI	Resting CD4+ helper T cells, naive CD4+ helper T cells, γδ T cells, memory B cells, naive B cells, plasma cells	RNA sequencing	Resting helper T cells^a^ • Healthy control: 13% • Trauma control: 4% • SCI: 3% Naive helper T cells^a^ • Healthy control: 14% • Trauma control: 12% • SCI: 8% γδ T cells^a^ • Healthy control: 0.1% • Trauma control: 1% • SCI: 2% B cells excluded

^a^ Deduced from graphics.

^b^ Restricted maximum likelihood (REML) units.

CNS, central nervous system; spinal cord injury.

### Effects of SCI on circulating B cells in humans

A loss of B cells or B cell dysfunction increases the risk for recurrent infections.^84^ B cells (CD19+, CD20+, CD79+)^85–88^ originate in the bone marrow and travel through the blood to home to lymphatic organs.^89^ B cells bind to activated helper T cells^90,91^ that recognize the same antigen and also function as antigen-presenting cells.^92^ In T cell-dependent B cell activation, T and B cells form an immunological synapse that triggers antigen-specific B cell clonal expansion.^90,93,94^ Some of these clones become long-lived memory B cells, whereas others become plasmablasts^95^ that differentiate into antibody-secreting plasma cells against one specific pathogen or self-antigen.^74,90,96^ Most plasma cells migrate to the bone marrow,^97^ and some home in lymph nodes.^98^ Differentiated B cells first secrete immunoglobulin (Ig)M antibodies^99^ and then class-switch to produce high-affinity IgG^100^ or IgA antibodies^97,100,101^ that bind to pathogens upon reexposure. Based on the SCI-IDS hypothesis, it is logical to believe that injury to the spinal cord reduces blood B cell numbers in the chronic phase of recovery. However, this is not the case.

To our knowledge, the acute effect of SCI on the number of blood B cells has only been studied in one population in Europe. Riegger and colleagues^47^ showed that the number of circulating CD19+ B cells decreases by >70% at 1 day after SCI and remains lower than baseline for 3–4 days compared with in people who had minor surgeries. However, this trend reverses a few days later when spinal cord injured people get blood B cell lymphocytosis and have 70% more B cells than people with minor surgeries.^47^ Circulating B cell counts return to baseline by 1 month after injury with little fluctuation thereafter.^47^

Like blood T cell lymphopenia, the initial blood B cell lymphopenia in humans is inversely proportional to neutrophilia.^44,45,47^ It is less understood why SCI causes B cell lymphopenia that progresses to lymphocytosis within the first week after SCI. Increased numbers of blood B cells are contradictory to SCI-IDS.

SCI does not affect blood B cell homeostasis in the chronic stages of recovery in humans regardless of injury level or severity (Table 1). The absolute count^71^ and ratio^69,70,80^ of B cells in the blood remain stable years after SCI, whether the injury is cervical,^69^ rostral to T5,^80^ or complete.^69,102^ Blood B cell homeostasis likely remains unchanged because injury to the spinal cord does not reduce B cell lymphopoiesis in people.^71^

Antigen-activated mature B cells differentiate into long-term plasma cells and memory cells to limit recurrent infections.^103^ Approximately 40% of adult B cells are memory B cells.^104^ Maturation into plasma cells leads to loss of CD19 and CD20 cell surface markers.^105,106^ B cell differentiation into plasma or memory cells after SCI has not been adequately studied but is, perhaps, more important than B cell homeostasis. SCI-IDS suggests loss of ability to fight infections. It is hence critical for the SCI field to understand if spinal cord trauma affects B cell differentiation into long-lived plasma or memory B cells because these cells are critical for long-term immunity.^107,108^

## Blood Lymphopenia in Rodents After SCI: Proceed with Caution

Lymphatic tissue atrophy,^42,43^ reduced bone marrow hematopoiesis,^32,42,43,49^ and reduced numbers of circulating lymphocytes^29^ observed in rodent studies have been offered as evidence to explain immunosuppression and, consequently, a high incidence of infections in humans after SCI.^29^ Grossly, the majority of circulating leukocytes in people are neutrophils, but in rodents, the majority of circulating leukocytes are lymphocytes.^109,110^ Researchers must carefully evaluate how to apply pre-clinical findings from rodent spinal cord trauma models to people with SCI because rodent and human immune systems differ significantly.^109,110^

Studies of immune function after SCI in rodents have used several injury models, such as complete transection, moderate contusion, and clip compression, and often animals are euthanized at each end-point to collect blood. The effect of SCI on blood lymphocyte homeostasis in rodents is species-, sex-, and injury model-specific (Table 2). In CD1 mice of unknown sex, the proportion of blood CD3+ T cells decreases significantly within 12 h but returns to normal at 96 h after T9/T10 contusion compared with sham-injured (laminectomy only) or uninjured mice.^111^ Male Lewis rats with spinal cord transection at T8 also lose 60% of total blood CD3+ T cells within 24 h, but the number of circulating CD3+ T lymphocytes is still low 2 weeks after injury compared with shams.^29^ Less is known about changes in circulating T cell numbers beyond 2 weeks after SCI. However, in female Wistar rats with C7/T1 clip compression, the percentage of blood CD3+ T cells does not differ significantly between injured rats and shams 2 weeks after surgery.^112^

**Table 2.** Manuscripts Comparing Blood Lymphocyte Responses in Rodents After SCI

Author	Subjects and injury type	Time frame	Cell type studied	Methods used	Results
Riegger et al. 2007	8 adult, male Lewis rats, *n* = 8, hemisection at spinal level T8 with fine iridectomy scissors; age-matched controls with laminectomy only	1, 3, 7, and 14 days after SCI	CD3+ T cells, CD45 RA + B cells	Flow cytometry	T cells^a^ • Control: 1481 • <24 h: 297 • 3-4 days: 597 • 6-8 days: 1652 • 25-30 days: 1966 • 105-136 days: 1771 B cells^a^ • Control: 256 • <24 h: 72 • 3-4 days: 133 • 6-8 days: 442 • 25-30 days: 373 • 105-136 days: 224
Stirling & Yong 2008	Adult CD-1 mice of unspecified sex contused at spinal level T9/T10 via Infinite Horizons Impactor (*n* = 3–5 mice for each time-point); naive, uninjured CD-1 mice and shams served as controls	12, 24, 48, and 96 h after SCI	CD3+ T cells	Flow cytometry	T cells • Naive: 29.0% • 12 h: 10.0% • 24 h: 34.3% • 48 h: 22.8% 96 h: 27.6%
Ulndreaj et al. 2020	111 adult, female Wistar rats injured at spinal level C7/T1 via clip compression; sham rats underwent C7-T1 laminectomy (*n* = 5–7 [2 weeks], 6 [10 weeks], 7 [20 weeks]) only	2 weeks, 10 weeks, and 20 weeks after SCI	CD4+ helper and CD8+ cytotoxic T cells, pan T or B cell detection unknown	Flow cytometry	T cells^b^: • 2 weeks: 12% (sham: 16%) • 10 weeks 15% (sham: 27%) • 20 weeks: 13% (sham: 13%) Helper T cells^b^: • 2 weeks: 68% (sham: 65%) • 10 weeks: 77% (sham: 78%) • 20 weeks: 61% (sham: 59%) Cytotoxic T cells^b^: • 2 weeks: 25% (sham: 32%) • 10 weeks: 19% (sham: 19%) • 20 weeks: 28% (sham: 30%) B cells^b^: • 2 weeks: 13% (sham: 13%) • 10 weeks: 12% (sham: 17%) • 20 weeks: 8% (sham: 8%)

^a^ In restricted maximum likelihood units (REML).

^b^ Deduced from graphics.

SCI, spinal cord injury.

Similar to changes in blood T cells, the number of blood CD45RA+ B cells in male Lewis rats decreases by >60% at 24 h and is lowest at 3 days after injury.^29^ Circulating B cell counts remain 40% lower in male Lewis rats at 2 weeks after spinal cord transection compared with sham injury.^29^ In contrast, in female Wistar rats, the percentage of blood B cells does not differ significantly at 2 weeks or 20 weeks after compression SCI compared with shams.^112^

Sex differences in incidence and rehabilitation outcomes after SCI have been recognized for decades. However, experiments with rodents that only include one sex dominate the SCI literature with a significant preference for females.^113^ Investigators typically exclude male rodents because bladder care of males after SCI is considered more difficult.^114^ Recent work by Ayala and colleagues^115^ showed that the peripheral blood leukocyte response to T9 contusion injury in Fischer-344 rats differs significantly between males and females. Because sex differences in immune responses correlate with sex differences in infarct size^116^ and recovery after brain trauma,^117,118^ future SCI research should also include males and females.

Blood lymphopenia in rodents^29^ may occur from splenic atrophy, clinically known as hyposplenism. The spleen is the largest lymphocyte reservoir in rodents.^119^ Elevated corticosterone and catecholamine levels after SCI correlate with splenic atrophy and lymphocyte apoptosis in mice.^42,43,49^ Lucin and associates^42,43^ showed that higher catecholamine and corticosterone concentrations, released after T3 but not T9 injury, correlate with splenic atrophy and lymphocyte apoptosis after SCI.

However, stress does not shrink the spleen^120^ and lymphocytosis, not lymphopenia, occurs after splenectomy in humans.^121,122^ Clinically, hyposplenism is diagnosed by the presence of erythrocytes with nuclear remnants, called Howell-Jolly bodies, clearly seen in simple blood smears.^123^ We did not find reports suggesting hyposplenism in people with SCI. Because spinal cord injured people typically undergo imaging such as magnetic resonance imaging (MRI) or computerized tomography scans to grade the lesion size and level, understanding if changes in spleen size correlate with SCI-IDS is a testable hypothesis. Without further evidence supporting clinical translation, blood lymphopenia from hyposplenism after SCI may be a rodent-specific phenomenon.

A more recent hypothesis to explain blood lymphopenia and increased infection risk in humans after SCI has been termed “bone marrow failure syndrome.”^32^ Based on studies in mice, it is thought that SCI impairs the development and maturation of leukocyte precursors in the bone marrow.^32^ However, it is not known if a decrease in bone marrow lymphocyte precursors occurs from mature “leukocyte reverse migration” to the bone marrow cavities,^49^ or if bone marrow hematopoiesis becomes dysfunctional, contributing to decreased blood lymphocyte counts in rodents.^29^ Further, whether SCI causes significant acute or chronic cellular changes in the bone marrow in humans remains controversial.^71,124^ Further studies should clarify whether SCI reduces the number of bone marrow cells or if bone marrow denervation causes cells to lose their proliferative capacity.

## Does SCI Reduce Blood Lymphocyte Function?

### Effects of SCI on blood lymphocyte function in humans

Pioneer hematological SCI studies concluded that spinal cord trauma decreases blood T and B cell function.^50,51,80^ Cruse and colleagues^50^ and Kliesch and associates^51^ collected blood from spinal cord injured people and healthy controls, isolated the mononuclear cell (MNC) layer, incubated MNCs with phytohemagglutinin/leucoagglutinin, a strong T cell mitogen,^125^ and examined the amount of DNA synthesis. They found that DNA synthesis in MNCs, from people at 3 months after SCI, is 30–40% lower than that in MNCs of healthy, uninjured people.^50,51^ Further, Herman and co-workers^80^ found that, compared to uninjured individuals, people with chronic SCI have reduced gene expression in foci related to T and B cells.

Other studies suggest that SCI does not reduce blood lymphocyte function in humans for several reasons. First, people with chronic SCI do not produce lower quantities of interleukin (IL)-2, which potentiates T cell division and differentiation,^126,127^ than healthy, uninjured controls.^128,129^ In fact, chronic quadriplegics have higher IL-2 receptor titers compared with people without SCI.^54,130^ Second, *cytomegalovirus* antigen and phorbol myristate acetate mitogen cause similar T cell activation (CD69 expression)^131^ and cell division (bromodeoxyuridine incorporation)^132^ in helper and cytotoxic T cells from healthy and spinal cord injured people.^133^ Third, some people with SCI have T cells that are more reactive to neural tissue proteins than T cells from people without SCI.^53^ Finally, even though intact T and B cell function are critical for vaccine-mediated immunity,^134,135^ antibody production to pneumococcal^136,137^ and influenza^138^ vaccines does not differ between people with and without SCI.

In contrast to the immune depression hypothesis, Saltzman and colleagues^52^ found that SCI increases gene expression related to immunological memory. They^52^ performed microarray and quantitative polymerase chain reaction on blood MNCs from mostly thoracic spinal cord injured people. They found that B cell maturation antigen (BCMA), a proliferation inducing ligand (APRIL), and B cell-activating factor (BAFF) are significantly upregulated in MNCs from spinal cord injured people compared with uninjured controls.^52^ BAFF and BCMA regulate B cell differentiation into plasma cells,^139^ and hence increased BAFF and BCMA suggest that SCI increases B cell differentiation into plasma cells. However, BAFF and APRIL expression is not B cell-exclusive and is also seen in monocytes, macrophages, dendritic cells, bone marrow stroma cells, and T cells.^140^ Without isolating blood B cells after SCI, it is difficult to assess the magnitude of BAFF and BCMA expression that is specific to this lymphocyte population.

Leukocyte isolation methods have become widely available, affordable, and require miniscule samples. Hence, future research should isolate individual blood lymphocyte populations to decipher the mechanism of SCI-IDS at the cellular level, especially because recent experiments in people suggest an increase in blood lymphocyte function after SCI. Although adaptive immune system function may be altered after SCI in humans, the activity of blood lymphocytes is likely not depressed.

### Effects of SCI on antibody synthesis

Antigen recognition leads to B cell differentiation into long-lived antibody-secreting plasma cells.^141^ Plasma cells secrete IgM, IgA, and IgG antibodies key to fighting the common infections that spinal cord injured people experience, such as pneumonia, influenza, and urinary tract infections (UTIs).^13,26,142–144^ IgM is the first antibody involved in the humoral immune response and protects the vasculature and, to a smaller extent, the mucosal surfaces,^144^ whereas IgA is the primary antibody involved in mucosal surface protection from pathogens.^142^ IgM and IgA thereby play key roles in fighting the respiratory infections and UTIs that spinal cord injured people frequently suffer.^13,26^

There are several IgG antibody subclasses, including IgG1, IgG2, IgG3, and IgG4.^145^ People with IgG1 and IgG2 deficiency are susceptible to bacterial capsule polysaccharide antigens such as the pneumonia-causing *Streptococcus pneumoniae*.^146–148^ IgG2 is highly selective for bacterial capsule polysaccharide antigens found on *S. pneumoniae*^149–151^ and *Haemophilus influenzae* type B.^150,151^ Reduced antibody synthesis could hence be one of the reasons why SCI increases the incidence of infections. However, evidence suggests that antibody synthesis remains functional in humans after SCI.

SCI reduces antibody synthesis in mice but not people. Shnawa and colleagues^152^ showed that people with chronic thoracolumbar or cervical SCI have increased IgG2 and IgA titers and normal IgG1, IgG3, IgG4, and IgM titers. In comparison, mice had lower titers of IgG1 in the first weeks after injury,^42,61^ but titers returned to normal during chronic recovery.^61^ Notably, IgG2 and IgA, the titers related to pneumonia and mucosal infections,^142,153^ are increased in people with SCI, yet IgG1, IgG3, IgG4, and IgM titers remain unchanged. This finding suggests that spinal cord injured people mount adequate antibody responses to common recurrent infections.

Clinical data also suggest that antibody synthesis remains functional in humans after SCI. Compared with healthy controls, people with SCI have a similar antibody titer response to *S. pneumoniae* and *H. influenzae* type B vaccines.^136–138^ Neither the time with chronic SCI nor the level of spinal cord damage affect the concentration of antibody titers after trivalent influenza or polyvalent pneumococcal vaccines.^136–138^ Further, people with SCI who were vaccinated against COVID-19 had similar rates of breakthrough infection compared with residents of skilled nursing facilities who were regularly tested for COVID-19.^154^

Despite SCI-IDS suggesting immunosuppression after SCI, researchers^53,61,128,129,155–158^ have also found significant autoantibody titers to injured spinal cord tissue fragments. Some spinal cord injured people^128,157^ have significantly higher immunoglobulin titers against myelin basic protein,^53,157^ glial fibrillary acidic protein,^156^ and GM_1_ ganglioside.^128,129^ Higher antibody titers against neural proteins may be seen days,^155,156,159^ weeks,^129,155,156,159^ and years after SCI.^53,128,129,157^ However, it is not well understood why not all people with SCI get autoantibodies against neural antigens^128,155,157^ nor why people without spinal cord trauma^155^ also have autoantibodies against central nervous system proteins.

Regulatory (suppressor) T cells (CD25+CD127lo) suppress immune responses against self-proteins,^160,161^ and autoimmunity occurs when regulatory T cells become dysfunctional. We expected to find that blood regulatory T cell function decreased after SCI. Loss of regulatory T cell function may explain why spinal cord injured people have increased autoantibody titers in the serum. However, Campagnolo and associates^69^ did not find significant differences in the ratio of helper to suppressor T cells between groups of people with and without cervical spinal cord injuries. Monahan and colleagues^72^ also showed that SCI does not significantly affect the proportion of regulatory T cells (CD25+CD127lo). In one cohort, those with SCI had higher percentages of blood CD4+CD25+CD127lo regulatory T cells that express C-C motif chemokine receptor 4 (CCR4), HLA-DR, or both CCR4 and HLA-DR compared with uninjured controls without a history of SCI.^72^ HLA-DR expression correlates with high suppressive activity of regulatory T cells,^72^ and CCR4 facilitates leukocyte migration.^72^ These data suggest that, although the numbers of blood regulatory T cells may remain unchanged, regulatory T cells in people with SCI are more able to migrate around the body and have a higher potential to suppress autoimmune responses than regulatory T cells in people without a history of SCI. Because this finding is contradictory to increased autoantibody titers, more studies are needed to understand how SCI causes autoimmunity.

## Discussion

Understanding how SCI affects acute and chronic immune responses to infections will improve clinical outcomes and reduce patient mortality. Researchers have proposed that a higher incidence of infections such as bacterial pneumonias,^6,7^ recurrent UTIs,^8,12^ and skin ulcer infections^9–11^ occurs because spinal cord trauma causes “spinal cord injury-induced immune depression syndrome” (SCI-IDS).^29^ SCI-IDS originates from studies in rodents that found lymphatic tissue atrophy,^42,43^ reduced bone marrow hematopoiesis,^32,42,43,49^ and blood lymphopenia.^29^ Because SCI-IDS is the leading hypothesis to explain higher infection rates in people with SCI, one would expect strong evidence of chronic blood lymphopenia and decreased lymphocyte function after SCI in people. However, a review of the literature showed that SCI in people and rodents leads to significant and species-specific changes in blood lymphocyte counts and function during the acute and chronic phases of recovery.

Spinal cord injured rodents and people have distinct blood granulocyte and lymphocyte responses. In rodents, transection injury of the spinal cord at thoracic and cervical levels causes the number of neutrophils to double or triple during the first days after injury^46,48^ and a profound blood T and B cell lymphopenia that correlates with splenic atrophy.^42,43^ Injuries in rodents at cervical and high thoracic levels cause more severe splenic atrophy.^42,43^ It remains unclear if and when blood lymphocyte counts recover after SCI in rodents.^29^

People with SCI also get significant blood neutrophilia^44,45,47^ that correlates with a significant increase in circulating pro-inflammatory proteins^128^ in the first days after trauma. Acute SCI-induced blood neutrophilia is also inversely correlated to the level of blood lymphopenia, and both return to baseline within the first week.^44,45,47^ Because blood neutrophils are the largest proportion of blood leukocytes (∼60%) in people,^110,111^ blood neutrophilia after SCI is not as pronounced compared with rodents.

The transient blood lymphopenia seen in spinal cord injured people^47^ may occur without the lymphatic organ atrophy seen in rodents. It would be useful to assess SCI-induced changes in lymphatic organs with MRI and blood smears during the acute and chronic stages of recovery. MRI is a routine clinical test used to assess spinal cord trauma,^162^ and functional asplenia causes a higher number of Howell-Jolly bodies seen in Wright-stained blood smears.^123^ These experimental approaches will help determine if the lymphatic atrophy seen in rodents translates to clinical practice.

Blood lymphocytes from people with SCI have mostly been quantified as percentages and seldom as absolute counts per microliter of blood. Immune responses to infections or immunosuppressive syndromes are tracked with absolute cell counts for several reasons. First, increased catecholamine and inflammatory responses^38–41^ cause immediate neutrophil demargination into the bloodstream,^44–48^ translating to an increased ratio of neutrophils to lymphocytes. Second, the absolute number of blood T cells per mm^3^ determines the infection prophylaxis treatment in cases of HIV; for example, people with < 200 CD4+ T cells/mm^3^ need trimethoprim/sulfamethoxazole prophylaxis, whereas people with < 50 CD4+ T cells/mm^3^ require azithromycin.^163^ Understanding changes in absolute blood leukocyte counts, in addition to percentages, will help determine the clinical approach to any potential immunosuppressive effects of SCI.

Despite the immune depression hypothesis, our review also found that blood T^53,54,128–133^ and B^52,152^ cells remain functional after SCI. People with SCI mount adequate immune responses to vaccines, producing similar antibody titers to encapsulated organisms that cause bacterial pneumonia^136,137^ compared with people with intact cords. Spinal cord injured people also produce more IgG2 and IgA^152^ antibodies to fight respiratory infections^142,143^ than people without SCI. In addition, if spinal cord trauma leads to immune depression, it is not clear why some people with SCI get higher autoantibody titers.^53,128,129,155–157^ Although gene expression studies^50–52,77,80^ found changes in blood leukocyte gene expression after SCI, future SCI studies with isolated T and B cell populations will shed additional light on the effects of SCI on adaptive immune cell function.

The lack of clinical translation of SCI-IDS is likely multi-factorial. First, there are significant species-specific differences in immune systems^110,111^ and immune responses after SCI.^29,60,112,113,164^ Second, rodent SCI studies of lymphocyte function were mostly done in splenic tissue, whereas all immune dysfunction assays in people have used blood samples. Third, it is difficult to conclude that SCI reduces adaptive immune function without quantifying blood T and B cells in absolute numbers over time or isolating cell functions in these leukocytes after cell sorting and multi-color flow cytometry.

So, why are people with SCI more likely to get infections with lethal consequences^13,20–23^ than people without SCI? Spinal cord injured people have more exposure to healthcare-associated infections^165–167^ and are at higher risk for pneumonia^168–170^ from aspiration or reduced lung mucociliary clearance.^24^ They are also more likely to need ventilator assistance,^24^ daily or recurrent urinary catheterization^25^ to treat urinary stasis from neurogenic bladder,^171^ and manual disimpaction to manage sphincter tone loss.^172,173^ Further, people with SCI primarily suffer from common bacterial infections such as pneumonia and UTIs,^26^ but not infections from opportunistic pathogens^174,175^ seen in people with dysfunctional immune responses or immune deficiencies.^176,177^ Continued research on the immune response to SCI and an emphasis on educating patients and providers on the prevention of common infections have the potential to significantly reduce infection rates in the future.
